# Supplementary material for: New Insights on the Feature and Function of Tail Tubular Protein B and Tail Fiber Protein of the Lytic Bacteriophage φYeO3-12 Specific for Yersinia enterocolitica Serotype O:3
Source: Molecules. 2020 Sep 24;25(19):4392. doi: 10.3390/molecules25194392 (PMC7582827; doi:10.3390/molecules25194392)

New insights on the feature and function of Tail Tubular Protein B and Tail Fiber Protein of the Lytic Bacteriophage φYeO3-12 specific for *Yersinia enterocolitica* Serotype O:3

Anna Pyra[1]*, Karolina Filik[2], Bożena Szermer-Olearnik[2], Anna Czarny[2], Ewa Brzozowska[2]**
[1] University of Wroclaw, Faculty of Chemistry, 14 F. Joliot-Curie St, Wroclaw, 50383, Poland
[2] Hirszfeld Institute of Immunology and Experimental Therapy, Polish Academy of Sciences, 12 R. Weigl St, Wroclaw, 53114, Poland
Correspondence to:
* anna.pyra@chem.uni.wroc.pl
** ewa.brzozowska@hirszfeld.pl

BLAST amino acid sequence analysis [28] showing similarity of TTPBgp12 to other TPs.

Phage Tail Protein (TP)	Phage of	% of amino acid sequence identity of TP
Tail Tubular Protein B (TTPB)	*Yersinia, Escherichia, Salmonella, Citrobacter, Serratia, Klebsiella, Leclercia, Enterobacteria*	97-99
	Salmonella, Serratia, Escherichia, Enterobacteria, Erwinia, Yersinia, Pectobacterium	65-70
	Pectobacerium, Erwinia, Pantoea, Yersinia, Escherichia, Enterobacteria, Salmonella, Klebsiella	62-65
Tail Fiber Protein (TFP)	*Enterobacter*	99-100
	Escherichia, Stenotrophomonas, Klebsiella	62-67

BLAST amino acid sequence analysis [29] showing similarity of TFPgp17 to other phage TFPs.

Phage of	% of amino acid sequence identity of TFP to TFPgp17
Enterobacter, Yersinia, Serratia, Citrobacter, Shigella, and *Escherichia*	99-100
Dickeya	92
Yersinia	81-84
Pseudomonas and *Klebsiella*	76 -78
Dickeya	68
Klebsiella, Escherichia, Stenotrophomonas and *Pseudomonas*	51-55
Leclercia, Escherichia, Pectobacterium, Erwinia, Enterobacteria, Yersinia, Xylella, Klebsiella, Serratia, Kluyvera and *Pseudomonas*	47-48
Pseudomonas, Serratia, Citrobacter and *Salmonella*	43-46
Pseudomonas	24

The BLAST amino acid sequence alignment scheme [28] showing similarity of TFPgp17 and TFP of *Enterobacteria* phage T7 (T7_TFP), TFP of *Enterobacteria* phage T3 (T3_TFP) of tail spike protein of *Enterobacteria* phage K1F (K1F_TSP), L-shaped TFP of *Escherichia* phage T5 (T5_TFP), side TFP of prophage K12 (K12_TFP) and TFP of *Enterobacteria* phage lambda (L_TFP).

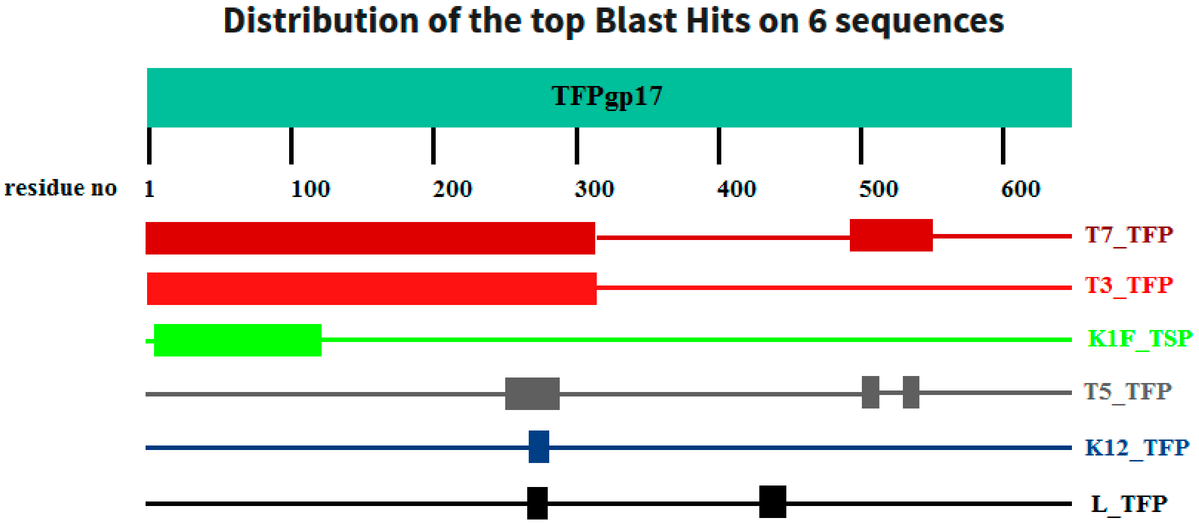

Sequence alignment of TTPBgp12 and T7_TTPgp12 from bacteriophage T7 (PDB code: 6r21) [13] using The Clustal Omega tool [24]. Identities between TTPBgp12 and T7_TTPgp12 are shown in red.

```
TTPBgp12      MALISQSIKNLKGGISQQPDILRFAEQGSVQINGWSSESEGLQKRPPMIHLKTLGPAGYV   60
T7_TTPgp12    MALISQSIKNLKGGISQQPDILRYPDQGSRQVNGWSSETEGLQKRPPLVFLNTLGDNGAL   60

TTPBgp12      GAQPYVHLINRDEFEQYFVVFTGEDIKVFDLDGKEYQVR--GDRSYVRTANPREDLRMIT   118
T7_TTPgp12    GQAPYIHLINRDEHEQYYAVFTGSGIRVFDLSGNEKQVRYPNGSNYIKTANPRNDLRMVT   120

TTPBgp12      VADYTFVTNRKVVVQSNDQSVNLPGFKDQGDALINVRGGQYGRRLSIEFNGAERAAVQLP   178
T7_TTPgp12    VADYTFIVNRNVVAQKNTKSVNLPNYNPNQDGLINVRGGQYGRELIVHINGKDVAKYKIP   180

TTPBgp12      DGSQPAHVNEVDGQAIAEKLAAQLRNNLGNPNNDQDPNKWRFNVGPGFIHILAPNNDNVW   238
T7_TTPgp12    DGSQPEHVNNTDAQWLAEELAKQMRTNL----------SDWTVNVGQGFIHVTAPSGQQID   231

TTPBgp12      GLQTKDGYADQLINPVTHYTQSFQKLPINAPDGYIVKIVGDTSKTADQYYVRFDLNRKVW   298
T7_TTPgp12    SFTTKDGYADQLINPVTHYAQSFSKLPPNAPNGYMVKIVGDASKSADQYYVRYDAERKVW   291

TTPBgp12      VETIGWNTRTHLYYHTMPWALVRASDGNFDFKVLEWGARTVGDDTTNPYPSFTGQTINDI   358
T7_TTPgp12    TETLGWNTEDQVLWETMPHALVRAADGNFDFKWLEWSPKSCGDVDTNPWPSFVGSSINDV   351

TTPBgp12      FFFRNRLGFLSGENIILSRTSKYFNFFPASVSNYSDDDPIDVAVSHNRVSTLKYAVPFSE   418
T7_TTPgp12    FFFRNRLGFLSGENIILSRTAKYFNFYPASIANLSDDDPIDVAVSTNRIAILKYAVPFSE   411

TTPBgp12      ELLLWSDQAQFVLTASGILSSRSVELNLTTQFDVQDRARPHGVGRNVYFASPRASFTSIN   478
T7_TTPgp12    ELLIWSDEAQFVLTASGTLTSKSVELNLTTQFDVQDRARPFGIGRNVYFASPRSSFTSIH   471

TTPBgp12      RYYAVQDVSSVKNAEDMTAHVPNYIPNGVFSISGTTAENFAAILTSGAPNRVYIYKFLYI   538
T7_TTPgp12    RYYAVQDVSSVKNAEDITSHVPNYIPNGVFSICGSGTENFCSVLSHGDPSKIFMYKFLYL   531

TTPBgp12      DEEIRQQSWSHWDFGDNVTVFAAQVINSTMTVLMGNEHAVWMGRLHFTKNSIDIPGEPYR   598
T7_TTPgp12    NEELRQQSWSHWDFGENVQVLACQSISSDMYVILRNEFNTFLARISFTKNAIDLQGEPYR   591

TTPBgp12      LYIDAKRKYTIPAGTYNDDTYQTSISLATIYGMNFTKGRVSVVFPDGKIIEVDQPINGWS   658
T7_TTPgp12    AFMDMKIRYTIPSGTYNDDTFTTSIHIPTIYGANFGRGKITVLEPDGKITVFEQPTAGWN   651

TTPBgp12      SDPVLRLDGNQEGQVVYIGFNIPFTYTFSKFLIKKTAEDGSTATEDIGRLQLRRAWVNYE   718
T7_TTPgp12    SDPWLRLSGNLEGRMVYIGFNINFVYEFSKFLIKQTADDGSTSTEDIGRLQLRRAWVNYE   711

TTPBgp12      DSGAFTIRVNNLSREFIYTMAGARLGSDNLRVGRSNIGTGQYRFPVVGNAQTNLVTIESD   778
T7_TTPgp12    NSGTFDIYVENQSSNWKYTMAGARLGSNTLRAGRLNLGTGQYRFPVVGNAKFNTVYILSD   771

TTPBgp12      ASTPLNIIGCGWEGNYLRRSSGI 801
T7_TTPgp12    ETTPLNIIGCGWEGNYLRRSSGI 794
```

The results of HHPred analysis of TFPgp17 [31].

No	Homologous template protein	Source	PDB code	% of probability	Amino acid residues of template protein	Amino acid residues of TFPgp17	The length of polypeptide chain
1	Endo-N-acetylneur-aminidase (chaperone)	*Enterobacteria* phage K1F	3GW6	99.4	10-275	419-643	208
2	Receptor recognition protein	*Salmonella* phage vB_SenMS16	6F45	99.3	78-193	524-645	113
3	L-shaped tail fiber protein	*Enterobacteria* phage T5	4UW8	99.2	232-426	412-645	156
4	Neck appendage protein (chaperone)	*Bacillus* phage GA-1	3GUD	99.1	1-123	539-641	93
5	phiAB6 tailspike	unidentified phage	5JSD	99.1	21-179	2-188	156
6	Tail spike protein	Acinetobacter phage vB_AbaP_AS12	6EU4	98.9	569-716	500-638	125

Multiple sequence alignment of TFPgp17 and TFP from phage T7 (T7_TFP, UniProt code: P03748) , TFP from phage T3 (T3_TFP, UniProt code: P10308), tail spike protein from phage K1F (K1F_TSP, UniProt code: Q04830). Identities between TFPgp17 and all other selected proteins are shown in dark blue; those between TFPgp17, T7_TFP andT3_TFP are shown in red; those between TFPgp17, T7_TFP and K1F_TSP are shown in grey; those between TFPgp17 and T7_TFP are shown in turquoise; those between TFPgp17 and T3_TFP are shown in cyan and those between TFPgp17 and K1F_TSP are shown in orange.

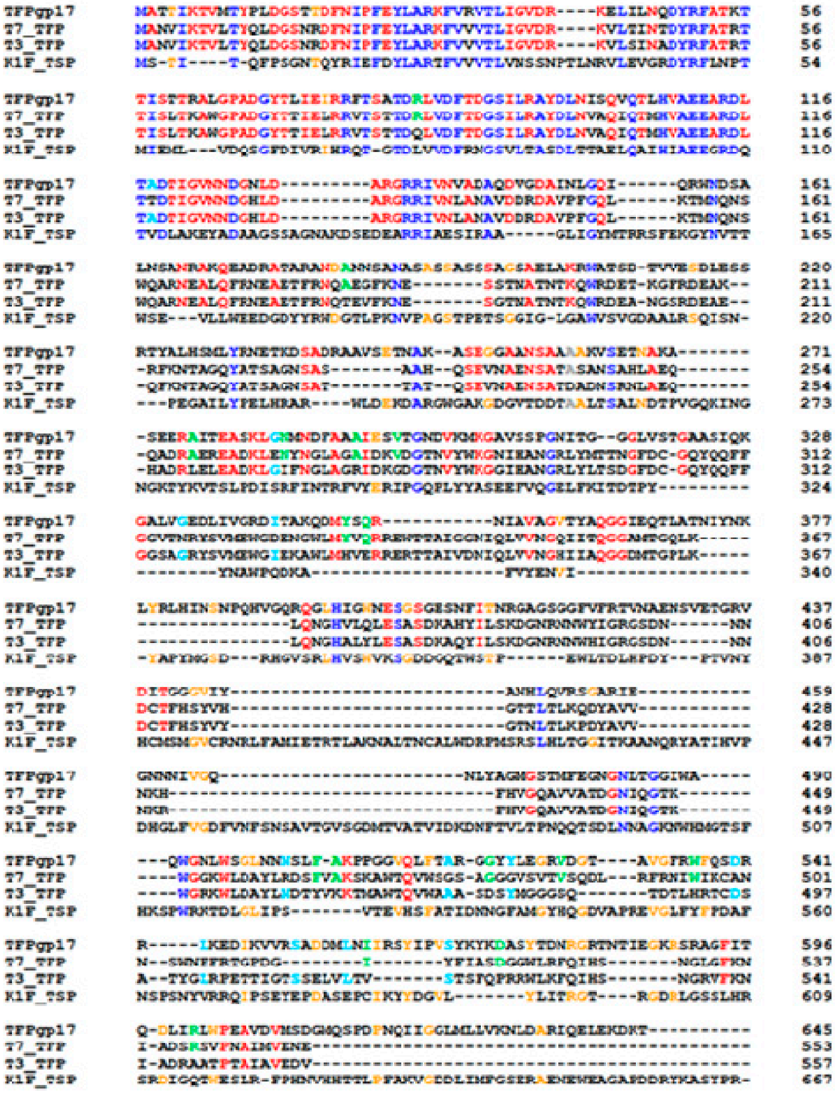

Multiple sequence alignment of TFPgp17 and L-shaped TFP from phage T5 (T5_TFP, UniProt code: P13390), side TFP from prophage K12 (K12_TFP, UniProt code: P76072) and TFP *from* phage lambda (L_TFP, UniProt code: P03764). Identities between TFPgp17 and all other selected proteins are shown in dark blue; those between TFPgp17, T5_TFP and K12_TFP are shown in turquoise; those between TFPgp17, K12_TFP and L_TFP are shown in red; those between TFPgp17, T5_TFP and L_TFP are shown in grey; those between TFPgp17 and T5_TFP are shown in pink; those between TFPgp17 and K12_TFP are shown in orange and those between TFPgp17 and L_TFP are shown in purple

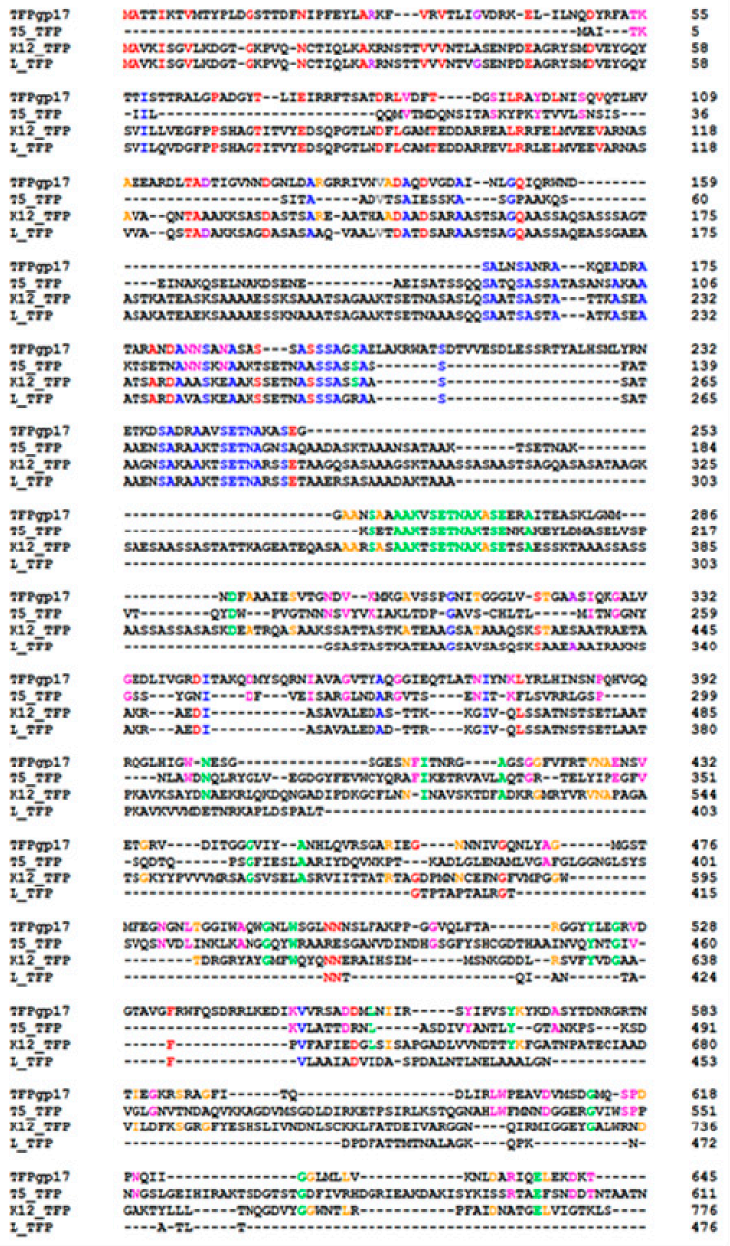

Supplement: Supplementary file 1 [file molecules-25-04392-s001.pdf]
